# Supplementary figures and images for: Phase II Study of Myeloablative 8/8- or 7/8-Matched Allotransplantation with Post-Transplant Cyclophosphamide, Tacrolimus, and Mycophenolate Mofetil: Marked Reduction in GVHD Risk Without Increased Relapse Risk Compared to Historical Cyclosporine/Methotrexate
Source: medRxiv. 2023 Mar 29:2023.03.24.23287521. Preprint. [Version 1] doi: 10.1101/2023.03.24.23287521 (PMC10081397; doi:10.1101/2023.03.24.23287521)

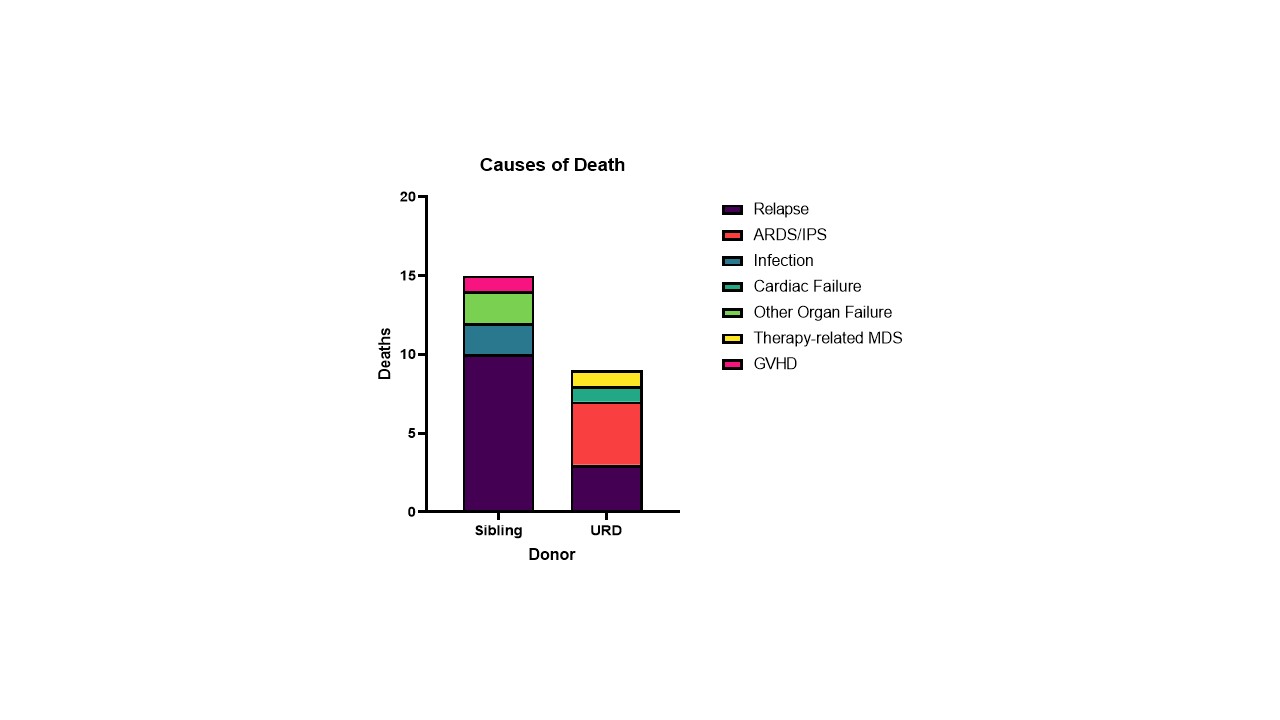

Supplement: Supplement 1 — Supplemental Figure 1: Causes of death by donor with PTCy/Tac/MMF GVHD prophylaxis. [file media-1.jpg]
